# Functional-Belief-Based Alcohol Use Questionnaire (FBAQ) as a Pre-Screening Tool for High-Risk Drinking Behaviors among Young Adults: A Northern Thai Cross-Sectional Survey Analysis

**DOI:** 10.3390/ijerph18041536

**Published:** 2021-02-05

**Authors:** Nalinee Yingchankul, Wichuda Jiraporncharoen, Chanapat Pateekhum, Surin Jiraniramai, Kanittha Thaikla, Chaisiri Angkurawaranon, Phichayut Phinyo

**Affiliations:** 1 Department of Family Medicine, Faculty of Medicine, Chiang Mai University, Chiang Mai 50200, Thailand; nalinee.j@cmu.ac.th (N.Y.); wichuda.j@cmu.ac.th (W.J.); chanapat_p@cmu.ac.th (C.P.); surin.j@cmu.ac.th (S.J.); chaisiri.a@cmu.ac.th (C.A.); 2 Research Institute for Health Sciences, Chiang Mai University, Chiang Mai 50200, Thailand; kanittha.th@cmu.ac.th; 3 Center for Clinical Epidemiology and Clinical Statistics, Faculty of Medicine, Chiang Mai University, Chiang Mai 50200, Thailand; 4 Musculoskeletal Science and Translational Research (MSTR) Cluster, Chiang Mai University, Chiang Mai 50200, Thailand

**Keywords:** beliefs, behavior, alcohol use, AUDIT, standard drink, screening, young adult

## Abstract

Background: an alcohol-use disorders identification test (AUDIT) is a standard screening tool for high-risk drinking behavior. Standard drink calculation is difficult to comprehend and may lead to inaccurate estimates. This study intended to develop a practical pre-screening tool for the identification of high-risk drinkers among young adults. Methods: a cross-sectional survey was conducted in Northern Thailand from July 2016 to December 2016. Data was collected on relevant characteristics and health beliefs about drinking. The 12-month AUDIT was used as the reference standard. Logistic regression was used for the score derivation. The discriminative ability was measured with an area under the receiver operating characteristic curve (AuROC). Result: a total of 1401 young adults were included. Of these, 791 people (56.5%) were current drinkers. Three functional-belief items were identified as independent predictors of high-risk drinking and were used to develop the functional-belief-based alcohol-use questionnaire (FBAQ). The FBAQ demonstrated an acceptable discriminative ability—AuROC 0.74 (95% confidence interval (CI) 0.70, 0.77). Conclusion: The FBAQ contains only three simple belief questions and does not require unintelligible standard drink calculation. Implementing the FBAQ score and the AUDIT in a serial manner might be a more effective method in a mass-screening program for alcohol-use disorder in young adults.

## 1. Introduction

Over the past decades, the worldwide prevalence of alcohol consumption has increased concurrently with alcohol-related problems and deaths [1], which raises significant public health concerns. A large Thai cohort study reported a great rise in the prevalence of alcohol consumption from 48% in males and 13% in females in 2007 [2] to 78% in males and 53% in females in 2017 [3]. Although the average alcohol consumption in Thailand was relatively lower than in the Western countries, evidence has shown that alcohol drinking would become more common over the years. Alcohol consumption patterns heavily influence the incidence of alcohol-related harm at both the individual and aggregated levels [4]. According to a cross-country analysis of the International Alcohol Control Study, high-frequency drinkers were usually found in high-income countries and older age populations. In contrast, heavy drinkers were usually found in middle-income countries and younger age populations [5]. Particular attention should be paid to the younger generation, as there have been consistent reports on decreasing age at onset of alcohol use [6,7,8] and that these groups were the ones that are at a higher risk of alcohol intoxication-related harms [9].

Harmful alcohol consumption and binge-drinking behavior among young adults, especially college students, results in various undesirable consequences, such as poor educational or work performance, detrimental effect on health and neurocognition, other substance use and drug overdoses, and death [10,11,12,13]. It is also widely recognized that drinking not only exposes harm to the drinkers but also to their surrounding social environments, which was due to drunk driving, gambling, sexual abuse, injuries, and domestic violence [14,15]. Thus, preventive measures to reduce harmful drinking are crucial. One approach is early identification and intervention of people who are at an increased risk of adverse outcomes from alcohol consumption through the screening, brief intervention, and referral to treatment (SBIRT) paradigm [16]. 

Several tools for screening of individuals with harmful alcohol-use behavior, including alcohol-use disorder (AUD), were developed, validated, and incorporated in local practice guidelines in many countries [17,18], such as the alcohol-use disorders identification test (AUDIT) and its abbreviated version, the alcohol use disorders identification test—consumption (AUDIT-C) [19,20]. The AUDIT was developed by the World Health Organization (WHO) to classify alcohol drinkers into four risk levels, which are low-risk, hazardous, harmful, and dependent drinkers, based on the frequency of drinking, the typical quantity consumed, and the frequency of heavy drinking. Then, a specific intervention (e.g., brief advice or referral for treatment) and health education is given for each risk level. Previous studies showed that the AUDIT demonstrated a strong correlation with Phosphatidylethanol (PEth), a direct alcohol biomarker [19,21]. AUDIT was suggested as an appropriate tool for evaluating alcohol use in young adults [17]. 

Despite its global popularity, the AUDIT carries one major limitation, which is a rather complex calculation of individual standard drinks, which requires a basic understanding of serving quantities and alcohol concentration by volume (ABV). The main difficulty in determining accurate standard drink is the variability of ABV within each beverage type, such as wine, beer, and spirits [22]. Furthermore, the definition of standard drink also varies across different countries as for the types of alcoholic beverage [23]. A previous study showed that drinkers often have difficulty defining their specific standard drinks and the estimated values usually underestimated the actual intake volume [22]. Thus, the method limits the standard drink as a theoretically accurate measure of quantity for alcohol consumption. Another important point lies within the practicality of the AUDIT. As the original version of the AUDIT contains a total of ten items, which is quite lengthy and time-consuming to evaluate when apply to a large body of population, a shortened version of AUDIT focusing on only consumption, the AUDIT-C was subsequently developed and had been increasingly popular [24,25]. However, the AUDIT-C still requires standard drink calculation. 

According to the health-belief model, a cognitive model of health behavior, cognition plays an important role in influencing individual health behavior, including health beliefs and substance use [26,27]. Both the beliefs about the substance itself and the beliefs about a person’s self-efficacy to control their substance use profoundly affect the severity of substance use [28,29]. Several studies showed that greater positive alcohol expectancies and lower self-efficacy are correlated with excessive alcohol consumption [30,31,32]. Specific health beliefs also influence alcohol use and the ability to giving up alcohol addiction, such as functional beliefs and risk-minimizing beliefs. Functional belief is the belief that the individual must rely on the use of alcohol to cope with daily life and duties [33], whereas risk-minimizing belief is the belief that the individual is less likely to be adversely affected by alcohol use [27]. These types of health beliefs commonly help drinkers to reduce their cognitive dissonance about negative consequences from alcohol [34]. One study examined the association between self-perception of associated health risk from alcohol consumption and the five-item AUDIT score and found that young people with higher AUDIT scores were unlikely to perceive that their drinking level was problematic, and that hazardous consequences of this level of use were acceptable to them [35]. Based on the results of our previous work, functional beliefs and risk-minimizing beliefs were associated with alcohol use and inversely associated with an intention to quit alcohol drinking [34]. To date, there is little evidence addressing the application of the health-belief model or health-belief intervention on alcohol-use disorder. Previously, a brief intervention based on the health-belief model was proven to increase self-awareness, improve understanding of physical harms due to alcohol addiction, and encourage quitting alcohol in hospitalized patients with alcohol-use disorders [36].

We hypothesized that the score from health-belief questions may be correlated with the AUDIT score and might be able to pre-screen young adults with high-risk drinking behavior without the need for complicated standard drink calculation. The primary aim of this study was to develop a simple health-belief based tool for pre-screening of individual with high-risk drinking behavior in the Northern Thai young adult population.

## 2. Materials and Methods

### 2.1. Study Design and Participants

This study was based on a cross-section household survey of the Northern Thai population, which was conducted from July 2016 to December 2016 using stratified multi-stage cluster random sampling design. The target population was local Thai residents aged between 12 to 65 years of age who were registered as residents within the Northern region according to the Department of Provincial Administration, the Ministry of Interior. All provinces in Northern Thailand were selected using systematic sampling with probability proportional to the size of the targeted population. Districts and households were selected in similar manners. From each household—the sampling unit— household members were stratified by gender, and were randomly selected using simple random sampling. Eligible participants must be able to communicate and answer the questionnaire, and must have resided within their residential communities and their households for more than six months and three months within the past year, respectively. As our study intended to explore the correlation between the health-belief score and the AUDIT score, and to develop a health-belief-based pre-screening tool for young adult populations, only participants aged between 18 to 39 years were included in the analysis. 

A total of 5920 eligible participants, aged between 12 to 65 years, from six provinces within the Northern region of Thailand were given informed consent and included in our primary cross-sectional survey data. After exclusion of participants in other age groups, 1401 young adults were included in the statistical analysis and model development. 

### 2.2. Data Collection

Each selected member was interviewed in a face-to-face manner with structured questionnaires by trained research personnel. Collected variables included demographic variables, socioeconomic variables (e.g., age, gender, personal income per month, education level, area of living, and occupation), attitudes towards substance use and self-experience, and health beliefs (both functional beliefs and risk-minimizing beliefs) for substance use. The 12-month AUDIT was applied to participants who had ever used alcohol and other substances within the past 12 months. The complete questionnaires were field checked by the research personnel after the interview. To minimize data entry errors, a double entry system was used. Comparative validation of the data was done with Epi info. Any discrepancy of data was corrected by reference to the primary questionnaire.

The assessment of health beliefs about alcohol drinking was evaluated using a nine-item questionnaire derived from previous studies [37,38]. The Thai version has been used and published in previous literature [34]. The first five items were regarding the individual functional beliefs of alcohol use, whereas the latest four items were regarding the individual risk-minimizing beliefs about the risk of alcohol use. Participants rated their agreement to each of the statement on a five-point-Likert scale from totally agreed, somewhat agreed, unsure, somewhat disagreed, and totally disagreed with the statement. A score was assigned to each question, ranging from 1 to 5, from total disagreement to total agreement, respectively. 

### 2.3. Study Endpoint

The primary outcome of interest was high-risk drinking behavior, which was determined from the 12-month AUDIT score [39]. According to previous literature, each participant can be categorized into four risk levels based on the total 12-month AUDIT score as follows: low-risk drinking (0–7), hazardous drinking (8–15), dangerous or harmful drinking (16–19), and almost certainly alcohol dependence (20 or more) [40,41]. In this study, we defined high-risk drinking behavior as participants who had a 12-month AUDIT score ≥8, which incorporates hazardous and harmful alcohol use and alcohol dependence. Participants who did not answer (never drank alcohol within the past 12 months), or who replied “never” or “less than monthly” were considered to have low-risk drinking behavior. 

### 2.4. Statistical Analysis

All statistical analysis and modeling were performed using Stata version 16 (StataCorp, College Station, TX, USA). Continuous variables were presented as mean and standard deviation, or median and interquartile range according to their distribution. Categorical variables were presented with frequency and percentage. Fisher’s exact probability test was used for comparison of categorical variables. Independent *t*-test and the Mann–Whitney U test was used for comparison of continuous variables as appropriate. Due to the ordinal nature of the data, Spearman’s rank correlation was used to evaluate the direction and the strength of a monotonic relationship between the health-belief score and the 12-month AUDIT score. 

#### 2.4.1. Diagnostic Score Derivation

Multivariable logistic regression was used for the derivation of the diagnostic model. Initially, all health-belief items were included in the multivariable model to explore significant predictors of high-risk drinking behavior. To reduce the number of predictors, a stepwise backward approach was used for the elimination of non-significant predictors with *p*-value < 0.05. From the final model, each of the remaining belief items were assigned with specific weighing based on their logit coefficients. To derive the weight, each log odds coefficient was divided by the smallest coefficient, and was subsequently rounded up to full integer. The crude score of each health-belief item, ranging from 1 to 5, would be multiplied by the assigned weight, and added up with the other remaining items to yield the product of the total health-belief-based score. The predicted probabilities of being high-risk drinkers for a set of given covariates (total score of final health-belief items) were estimated from the final logistic model and visualized with risk curves. 

#### 2.4.2. Diagnostic Performance of the Score

The test of performance was done in two aspects-discrimination, and calibration. The discriminative ability was quantified with the use of area under receiver operating characteristic curve (AuROC). In terms of model calibration, two methods were performed at each discrimination point. First, Hosmer and Lemeshow’s goodness-of-fit test was used to evaluate model fitting at a 0.1 significance level. Second, the calibration plot was presented by contrasting the model-predicted probabilities and the observed proportions of being in the higher alcohol-consumption risk level. Internal validation of the model discrimination was done via a bootstrap resampling procedure with 500 replicates. The whole modeling process was done within each bootstrap loop to examine the model optimism and overfitting.

#### 2.4.3. Diagnostic Accuracy of the Score

The sensitivity, specificity, positive likelihood ratio, and negative likelihood ratio of the newly-derived health-belief score for discriminating participants with high-risk drinking behavior from participants with low-risk drinking behavior at three cut-off points were examined using the 12-month AUDIT score at the cut-off point of 8 as the reference standard. All analyses were also done separately for males and females to explore for possible effect modification. 

## 3. Results

The mean age of the participants was 29.9 ± 6.3 years, with slightly male predominance (763, 54.5%). The remaining detail on other relevant characteristics of the included participants is shown on Table 1.

About 78.9% of male participants and 29.6% of female participants reported alcohol use within the past 12 months. The number of smokers was much lower than the number of drinkers in this survey (Table 1). The average 12-month AUDIT score for male participants was 4.8 (SD 4.5, Median 4 (IQR 1, 7), range 0–24) whereas for female participants, the average 12-month AUDIT score was 1.2 (SD 2.8, Median 0 (IQR 0, 1), range 0–24). According to the 12-month AUDIT evaluation, 1188 (84.8%) were low-risk or non-drinkers, 185 (13.2%) were hazardous drinkers, 19 (1.4%) were harmful drinkers, and 9 (0.6%) were alcohol dependent. The proportion of high-risk drinkers (12-month AUDIT ≥ 8) was substantially higher in males than in females (24.0% vs. 4.7%, *p* < 0.001).

**Figure 1:**
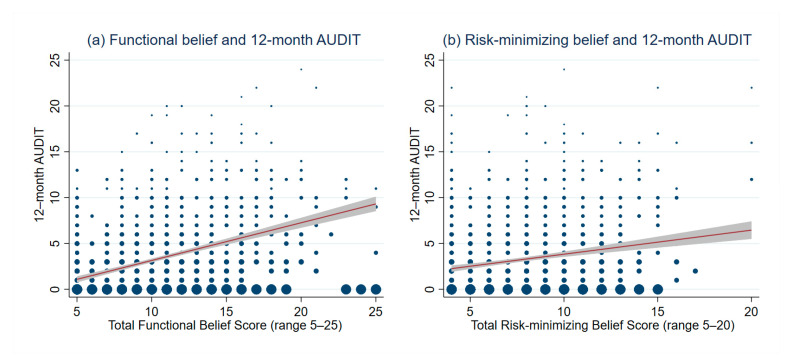
Scatter plots visualizing correlation between health beliefs and 12-month alcohol-use disorders identification test (AUDIT) score. (**a**) Monotonic correlation between functional-belief score and 12-month AUDIT score, (**b**) monotonic correlation between risk-minimizing belief score and 12-month AUDIT score.

Differences of functional-belief score and risk-minimizing belief score concerning the alcohol use between participants with high-risk drinking behavior and participants with low-risk drinking behavior was shown on Table 2. The mean functional-belief scores in high-risk drinkers and low-risk drinkers were significantly different in both males (12.8 ± 4.5 vs. 10.1 ± 3.7, *p* < 0.001) and females (14.3 ± 3.5 vs. 9.0 ± 3.6, *p* < 0.001). The mean risk-minimizing belief scores in high-risk drinkers and low-risk drinkers was also significantly different in both males (8.3 ± 3.6 vs. 7.4 ± 2.7, *p* < 0.001) and females (10.9 ± 3.1 vs. 7.2 ± 2.7, *p* < 0.001). Significant positive correlations were identified between the functional-belief score and the total 12-month AUDIT score (Spearman’s rho 0.38, *p*-value < 0.001) (Figure 1a). The same pattern was also observed between the risk-minimizing belief score and the 12-month AUDIT score (Spearman’s rho 0.13, *p*-value < 0.001) (Figure 1b).

From, multivariable logistic regression, only the first, the second and the fifth item of the function beliefs score were significantly associated with high-risk drinking behavior (Table 2). After stepwise elimination of non-significant predictors, the final three remained within the final parsimonious model (Table 3). The strongest predictor was the second item, which stated “Alcohol drinking calms you down when you are stressed or upset”. However, the point estimates and confidence intervals of association among all three items were highly overlapped. Thus, equal weight of one was assigned to each of the item. As each item was in the five-point Likert scale, the sum of the newly derived three-item score would range from 3 to 15.

**Figure 2:**
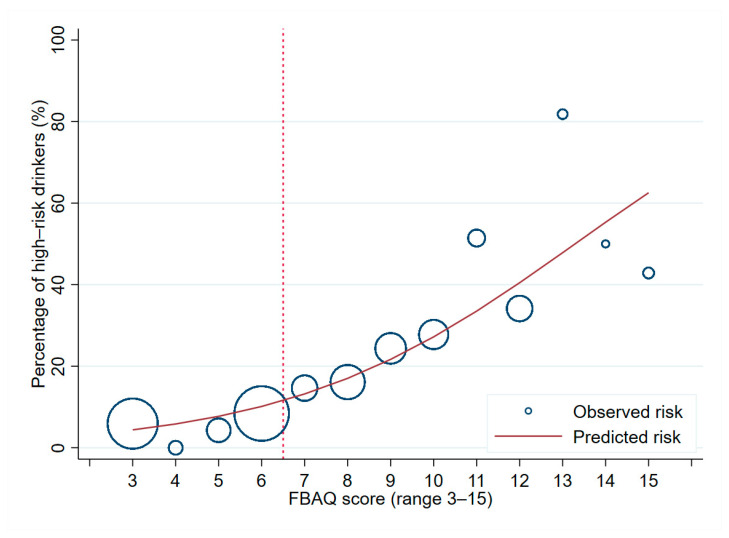
Calibration plot visualizing the agreement between the functional-belief-based alcohol-use questionnaire (FBAQ) score and the probability of being a high-risk drinker. Predicted-risk curve (red line) and observed risk (hollow blue circle) are shown, when the 12-month AUDIT was used as reference standard.

The mean estimated score was significantly higher in the high-risk drinking group compared to the low-risk group (8.8 ± 3.0 vs. 6.3 ± 2.7, *p* < 0.001). The mean scores in high-risk drinkers and low-risk drinkers were also significantly different in both males (8.7 ± 3.0 vs. 6.8 ± 2.8, *p* < 0.001) and females (10.0 ± 2.5 vs. 5.9 ± 2.5, *p* < 0.001). The three-item functional-belief score was able to distinguish low-risk drinkers from higher-risk drinkers at an AuROC of 0.74 (95%CI 0.70, 0.77), which was considered acceptable discriminative ability according to Hosmer and Lemeshow. The probability of being a high-risk drinker for each score point was estimated and visualized in predicted-risk curves (Figure 2). From this figure, it was observed that the probability of being in the high-risk drinking group increased as the three-item score increased. In terms of calibration, the Hosmer–Lemeshow goodness-of-fit test showed a non-significant *p*-value (*p* = 0.186). The model calibration was also presented in the risk curve (Figure 2). Internal validation via bootstrap resampling procedure yielded minimal AuROC optimism of +0.000261 (minimum-0.07004, maximum-0.047077). Detailed reports on internal validation were available in the appendix (Supplementary Table S2).

The diagnostic indices of the newly derived three-item score against the 12-month AUDIT at three different cut-off points are shown in Table 4. At the cut-off point of ≥6, the overall sensitivity and specificity of the score was 89.7% (95% CI 84.8, 93.4) and 33.0 (30.3, 35.8), respectively. No false negative of harmful drinkers and alcohol dependence was identified at the cut-off point below or equal to 6, whereas there was about 10 percent false negative at the cut-off point above or equal to 7 (Supplementary Table S1). For concurrent validity among genders, both the sensitivity and the specificity of the three-item score were on average higher in females than in males (Table 4). However, there were no significant differences between genders in sensitivity for detecting high-risk drinking (88.5% vs. 96.7%, *p* = 0.204) at the cut-off point of ≥6. 

## 4. Discussion

In this study, we have identified the positive correlation between the 12-month AUDIT score and both types of health-belief score (i.e., functional-belief and risk-minimizing belief score), which confirms our prior hypothesis. After multivariable modeling, three functional-belief questions were found to be significantly associated with high-risk drinking behavior in young adult participants, and the model discriminative ability was acceptable. These three, simple functional-belief questions, entitled functional-belief-based alcohol use questionnaire or the FBAQ, could be a potential pre-screening tool prior to the conventional AUDIT to rule out low-risk drinkers without the need for a complicated standard drink evaluation.

Previous studies had consistently identified the influential effect of individual health beliefs on alcohol drinking behavior [26,42,43]. Although drinkers usually perceive that their current behaviors might lead to negative consequences, they often convert their thought into positive ways. Unsurprisingly, high-risk drinkers were found to have significantly higher functional beliefs and risk-minimizing belief scores, which indicates that the health-belief scores might be able to discriminate high-risk drinkers from low-risk drinkers. Interestingly, the functional-belief items showed a significantly higher predictive impact than the risk-minimizing-belief items in identifying high-risk drinkers. This might be explained with two reasons. Firstly, alcohol consumption directly stimulates the release of dopamine and serotonin, which reduces stress, and promotes socializing function and merriment [44,45], which is in concordance with the three functional-belief questions of the FBAQ. The association between functional beliefs and alcohol use may be more prominent in young adult populations than older populations, as they usually use alcohol drinking as a mean to socialize and gain acceptance from friends and their social groups [46,47,48]. Secondly, risk-minimizing beliefs are quite fragile and can be altered easily by providing negative information and appropriate feedback [34].

For practicality, three potential cut-off points were examined using diagnostic indices. To create a pre-screening tool for AUDIT, we focused mainly on the sensitivity of the FBAQ. Based on our results, an FBAQ score ≥ 6 can be used as the optimal cut-off point for pre-screening of high-risk drinkers in young adult population. At this cut-off point, the sensitivity was high, and no participants with harmful drinking or alcohol dependence were missed. In other words, harmful drinking and alcohol dependence can be ruled out in respondents with an FBAQ score < 6. However, we suggested that regular health promotional information on appropriate alcohol consumption should still be given to all young adults regardless of their initial risk evaluation. For respondent with an FBAQ score ≥6, the standard AUDIT should be applied to categorize them into risk groups to guide a proper management plan. 

Interestingly, the sensitivity of the FBAQ score at all three cut-off points were higher in the female participants than those of the male participants, which suggests a potential modification effect of gender on the screening accuracy of the FBAQ score. Regarding substance use and addiction, both biological differences and sociocultural influences play important roles [49]. According to our data, there was a clearer difference of health beliefs between those who drank alcohol and those who did not in females than in males. Alcohol use in females was also less likely to be influenced by other factors beyond individual health beliefs as in males, such as social and peer pressure [50,51]. However, the modifying effect of gender on the sensitivity of the FBAQ score was not a statistically significant difference. Therefore, the screening of high-risk drinking from the FBAQ can be applied in both genders. Further study might focus on different components of health belief that affect alcohol drinking behavior in each gender.

The newly derived FBAQ score carries two major strengths. Firstly, the FBAQ score was derived from a household cross-sectional survey of the Northern Thai population with rigorous cluster sampling scheme and data management plan. Thus, the study base was a proper representative for the young adult population within the region. Secondly, the score carries high applicability with only three comprehensible questions and does not require complicated standard drink evaluation, which has hindered the efficient use of the AUDIT in a large population. By applying the FBAQ score as a pre-screening tool, the screening process within the SBIRT paradigm could be faster and more effectively delivered.

There were some limitations of the FBAQ to be addressed. First, the FBAQ score is a subjective measurement. It is likely and possible that the association between functional beliefs and alcohol drinking behavior differs across individuals with different backgrounds. However, the score was derived from large, multiprovincial survey data with heterogenous population, which enhances both the validity and the generalizability of the score. In addition, the association between functional beliefs and substance use was also supported by several studies [31,34,37,52]. Second, the 12-month AUDIT as the standard reference for identifying high-risk drinking behaviors could be considered subjective compared to the use of direct alcohol biomarker such as phosphatidylethanol (PEth). However, several studies had demonstrated strong correlation and good agreement between the AUDIT assessment and PEth [21] and suggested the use of AUDIT as a potentially more cost-effective tool for discrimination of high-risk and low-risk drinkers compared to a more costly PEth [19]. Third, the derivation set was composed of only young adult populations in the Northern region of Thailand, who participated the survey in 2016. Thus, we were aware that the FBAQ score might not be applicable for people in other age groups, or people who live in other regions or other countries, until the transportability of the score was confirmed in a further domain validation study. Finally, although the discriminative ability was shown to be acceptable and robust during an internal validation, a prospective external validation study is necessary prior to the implementation of the FBAQ score in practice. 

## 5. Conclusions

In conclusion, excessive alcohol drinking in young adults is an emerging global public health problem as it increases the risk of adverse health outcomes and social effects to both the individuals and their surrounding social environments. Early identification of people with high-risk drinking behavior through screening is a common approach to reduce adverse consequences of inappropriate alcohol use. In this study, we have developed a functional-belief-based assessment tool, called FBAQ, to be used as a simple pre-screening tool for young adults with high-risk drinking behavior. Unlike the standard AUDIT, the FBAQ does not require unintelligible standard drink calculation. It contains only three simple belief questions, which can be self-administered with ease. The respondents rate their agreement with the given statement on a score range of 1 to 5, from total disagreement to total agreement, for each question. Respondents with an FBAQ score less than 6 would be considered negative and are unlikely to be high-risk drinkers. Respondents with a positive FBAQ score, or FBAQ ≥ 6, still require confirmatory evaluation with the AUDIT. We believed that implementing the FBAQ score and the AUDIT in a serial manner might be a more effective way in a mass-screening program for alcohol-use disorder in young adults. This approach would undoubtedly decrease unnecessary AUDIT evaluations in young adults with low-risk drinking behavior, saving time and resources. 

## Figures and Tables

**Table 1:** Characteristics of study participants (n = 1401).

Characteristics	Missing Data	Male (n = 763)		Female (n = 638)		Overall (n = 1401)	
	n (%)	n	(%)	n	(%)	n	(%)
Age (years, mean ± SD)	0(0)	29.6	±6.4	30.2	±6.1	29.9	±6.3
Religion							
Buddhism	1(0.1)	742	(97.4)	612	(95.9)	1354	(96.7)
Others		20	(2.6)	26	(4.1)	46	(3.3)
Marital status							
Single/divorced	8(0.6)	360	(47.6)	186	(29.3)	546	(39.2)
Married		397	(52.4)	450	(70.7)	847	(60.8)
Occupation							
Constant income	0(0)	108	(14.2)	70	(11.0)	178	(12.7)
Non-constant income		503	(65.9)	400	(62.7)	903	(64.5)
No income/vacancy		47	(6.2)	63	(9.9)	110	(7.9)
Others/unspecified		105	(13.8)	105	(16.5)	210	(15.0)
Current smoker (within 12 months)	0(0)	298	(37.9)	11	(1.7)	300	(21.4)
Current drinker (within 12 months)	0(0)	602	(78.9)	189	(29.6)	791	(56.5)
Last drinking							
Within 1 week	0(0)	326	(42.7)	80	(12.5)	406	(29.0)
Within 1 month		178	(23.3)	61	(9.6)	239	(17.1)
Over 1 month		98	(12.8)	49	(7.7)	147	(10.5)
Over 12 months or never drink		161	(21.1)	448	(70.2)	609	(43.4)
Drinking frequency within the past 12 months							
Never drink	0(0)	161	(21.1)	450	(70.5)	611	(43.6)
5–7 days per week		233	(30.5)	134	(21.0)	367	(26.2)
3–4 days per week		142	(18.6)	28	(4.4)	170	(12.1)
1–2 days per week		128	(16.8)	16	(2.5)	144	(10.3)
<4 times per month		99	(13.0)	10	(1.6)	109	(7.8)
Number of standard drinks per day							
Never drink	0(0)	315	(41.3)	537	(84.2)	852	(60.8)
1–2		264	(34.6)	66	(10.3)	330	(23.6)
3–4		111	(14.6)	26	(4.1)	137	(9.8)
5–6		38	(5.0)	4	(0.6)	42	(3.0)
7–9		35	(4.6)	5	(0.8)	40	(2.9)
≥10							
Total AUDIT score (mean ± SD)	0(0)	4.8	±4.5	1.2	±2.8	3.2	±4.2
Median (IQR)	0(0)	4	(1, 7)	0	(0, 1)	1	(0, 5)
0–7	0(0)	580	(76.0)	608	(95.3)	1188	(84.8)
8–15		158	(20.7)	27	(4.2)	185	(13.2)
16–19		17	(2.2)	2	(0.3)	19	(1.4)
20–40		8	(1.1)	1	(0.2)	9	(0.6)
High-risk drinking (≥8)		183	(24.0)	30	(4.7)	213	(15.2)
Could not stop drinking	611(43.6)	71	(11.8)	20	(10.6)	91	(11.5)
Impaired function due to drinking	610(43.5)	27	(4.5)	16	(8.5)	43	(5.4)
Drinking for activity of daily living	610(43.5)	20	(3.3)	6	(3.2)	26	(3.3)
Attempt to quit alcohol drinking							
Never	461(32.9)	538	(82.6)	258	(89.3)	796	(84.7)
Within the last 3 months		45	(6.9)	17	(5.9)	82	(8.7)
More than 3 months		68	(10.5)	14	(4.8)	62	(6.6)

Abbreviations: SD, standard deviation; IQR, interquartile range; AUDIT, alcohol-use disorders identification test.

**Table 2:** Differences in functional beliefs and risk-minimizing beliefs between two risk groups.

	High-Risk Drinking (AUDIT ≥ 8) (n = 213)		Low-Risk Drinking (AUDIT ≤ 7) (n = 1188)			Multivariable Model		
	Mean	±SD	Mean	±SD	*p*-Value	OR	95%CI	*p*-Value
Functional beliefs								
(1) You enjoy alcohol drinking too much to give it up	2.49	±1.23	1.76	±0.87	**<0.001**	1.25	1.04, 1.52	**0.020**
(2) Alcohol drinking calms you down when you are stressed or upset	3.08	±1.27	2.12	±1.14	**<0.001**	1.37	1.14, 1.64	**0.001**
(3) Alcohol drinking helps you concentrate better	2.13	±1.12	1.63	±0.74	**<0.001**	0.96	0.74, 1.25	0.763
(4) Alcohol drinking is an important part of your life	2.11	±1.06	1.59	±0.71	**<0.001**	1.25	0.96, 1.63	0.094
(5) Alcohol drinking makes it easier for you to socialize	3.28	±1.24	2.45	±1.24	**<0.001**	1.25	1.06, 1.47	**0.007**
Risk-minimizing beliefs								
(6) The medical evidence that alcohol drinking is harmful is exaggerated	2.54	±1.29	2.26	±1.20	**0.002**	1.05	0.88, 1.24	0.599
(7) Alcohol drinking is no riskier than lots of other things that people do	2.21	±1.19	1.86	±0.94	**<0.001**	1.08	0.86, 1.36	0.507
(8) You have got to die of something, so why not enjoy yourself and alcohol drinking	1.97	±1.05	1.61	±0.67	**<0.001**	0.93	0.71, 1.21	0.574
(9) You have the kind of genetic makeup that allows you to drink without it giving your health problems	1.97	±1.01	1.60	±0.66	**<0.001**	1.23	0.96, 1.58	0.104
Total beliefs score	21.77	±6.37	16.89	±5.47	**<0.001**			

Abbreviations: SD, standard deviation; OR, odds ratio; CI, confidence interval. Statistically significant *p*-values are shown in bold.

**Table 3:** Final multivariable model after stepwise backward elimination of non-significant predictors of high-risk drinking behavior. (n = 1401).

	OR	95% CI	*p*-Value	Coefficient	Weighted Score
You enjoy alcohol drinking too much to give it up	1.36	1.15, 1.62	**<0.001**	0.3101	1 × score
Alcohol drinking calms you down when you are stressed or upset	1.40	1.17, 1.66	**<0.001**	0.3331	1 × score
Alcohol drinking makes it easier for you to socialize	1.29	1.10, 1.50	**0.001**	0.2512	1 × score
Constant	0.02			−3.9496	

Abbreviations: OR, odds ratio; CI, confidence interval. Statistically significant *p*-values are shown in bold.

**Table 4:** Comparative diagnostic accuracy of the FBAQ score with a reference standard (12-month AUDIT).

Reference Standard	Index Test	Sensitivity (95% CI)	Specificity (95% CI)	Positive Likelihood Ratio (95% CI)	Negative Likelihood Ratio (95% CI)
Overall (n = 1401)					
12-month AUDIT ≥ 8	FBAQ ≥ 5	91.1 (86.4, 94.5)	27.4 (24.8, 30.0)	1.25 (1.19, 1.32)	0.33 (0.21, 0.51)
	FBAQ ≥ 6	89.7 (84.8, 93.4)	33.0 (30.3, 35.8)	1.34 (1.26, 1.42)	0.31 (0.21, 0.47)
	FBAQ ≥ 7	74.6 (68.3, 80.3)	62.4 (59.5, 65.1)	1.98 (1.78, 2.21)	0.41 (0.32, 0.51)
Male (n = 763)					
12-month AUDIT ≥ 8	FBAQ ≥ 5	90.2 (84.9, 94.1)	21.6 (18.3, 25.1)	1.15 (1.08, 1.23)	0.46 (0.29, 0.73)
	FBAQ ≥ 6	88.5 (83.0, 92.8)	27.6 (24.0, 31.4)	1.22 (1.14, 1.31)	0.42 (0.27, 0.64)
	FBAQ ≥ 7	72.7 (65.6, 79.0)	54.5 (50.3, 58.6)	1.60 (1.41, 1.81)	0.50 (0.39, 0.64)
Female (n = 638)					
12-month AUDIT ≥ 8	FBAQ ≥ 5	96.7 (82.8, 99.9)	32.9 (29.2, 36.8)	1.44 (1.32, 1.57)	0.10 (0.01, 0.70)
	FBAQ ≥ 6	96.7 (82.8, 99.9)	38.2 (34.3, 42.2)	1.56 (1.43, 1.71)	0.09 (0.01, 0.60)
	FBAQ ≥ 7	86.7 (69.3, 96.2)	69.9 (66.1, 73.5)	2.88 (2.39, 3.47)	0.19 (0.08, 0.48)

Abbreviations: AuROC, area under the receiver operating characteristic curve; CI, confidence interval; AUDIT, alcohol-use disorders identification test; FBAQ, functional-belief alcohol questionnaire.

## Data Availability

The datasets used and/or analyzed during the current study are available from the corresponding author on reasonable request.

## Supplementary Materials

The following are available online at https://www.mdpi.com/1660-4601/18/4/1536/s1, Table S1: accuracy in classification of the FBAQ score with a reference standard (12-month AUDIT) at three specific cut-off points, Table S2: internal validation of discriminative performance with bootstrap resampling procedures for two discrimination points.
