# Supplementary material for: Functional-Belief-Based Alcohol Use Questionnaire (FBAQ) as a Pre-Screening Tool for High-Risk Drinking Behaviors among Young Adults: A Northern Thai Cross-Sectional Survey Analysis
Source: Int J Environ Res Public Health. 2021 Feb 5;18(4):1536. doi: 10.3390/ijerph18041536 (PMC7915812; doi:10.3390/ijerph18041536)
Supplement: Supplementary file 1 [file ijerph-18-01536-s001.pdf]

Supplementary information

Supplementary Table S1 accuracy in classification of the FBAQ score with a reference standard (12-month AUDIT) at three specific cut-off points.

Classification	Low-risk drinkers	Hazardous drinkers	Harmful drinkers	Alcohol dependence	Total
	n (%)	n (%)	n (%)	n (%)	n (%)
FBAQ					
<5	325 (27.4)	19 (10.3)	0 (0)	0 (0)	344 (24.6)
≥5	863 (72.6)	166 (89.7)	19 (100)	9 (100)	1057 (75.4)
Total	1188 (100)	185 (100)	19 (100)	9 (100)	1401 (100)
FBAQ					
<6	392 (33.0)	22 (11.9)	0 (0)	0 (0)	414 (29.6)
≥6	796 (67.0)	163 (88.1)	19 (100)	9 (100)	987 (70.4)
Total	1188 (100)	185 (100)	19 (100)	9 (100)	1401 (100)
FBAQ					
<7	741 (62.4)	51 (27.6)	2 (10.5)	1 (11.1)	795 (56.8)
≥7	447 (37.6)	134 (72.4)	17 (89.5)	8 (88.9)	606 (43.2)
Total	1188 (100)	185 (100)	19 (100)	9 (100)	1401 (100)

Supplementary Table S2 internal validation of discriminative performance with bootstrap resampling procedures for two discrimination points.

Variable	Replicates	Mean	Standard deviation	Minimum	Maximum
Discrimination of low risk drinking from hazardous/dangerous drinking					
Apparent AuROC	500	0.735894	0.019344	0.66556	0.78268
Apparent slope	500	6.335896	0.330747	5.270543	7.388781
Test AuROC	500	0.735603	0	0.735603	0.735603
Test slope	500	0.999614	0.049161	0.89447	1.176537
Optimism AuROC	500	0.000291	0.019344	-0.07004	0.047077
Optimism slope	500	0.000386	0.049161	-0.17654	0.10553
